# A115 THE PERFORMANCE OF NATURAL LANGUAGE PROCESSING IN INTERPRETING COLONOSCOPY REPORTS: A SYSTEMATIC REVIEW AND META-ANALYSIS

**DOI:** 10.1093/jcag/gwac036.115

**Published:** 2023-03-07

**Authors:** N Sabrie, R Jogendran, R Khan, M Scaffidi, N Gimpaya, D Lightfoot, S Grover

**Affiliations:** 1 University of Toronto; 2 Gastroenterology; 3 St. Michael's Hospital; 4 Gastroenterology, University of Toronto, Toronto, Canada

## Abstract

**Background:**

Screening colonoscopy is integral in the effort to identify and remove potentially cancerous lesions. Important quality indicators include the adenoma detection rate and more recently, the sessile/serrated adenoma detection rate. Natural language processing (NLP) is a computer-based linguistic technique that leverages artificial intelligence to abstract meaningful information from text. This tool carries the potential to automate the task of analyzing large volumes of colonoscopy and pathology reports to generate data on key performance metrics.

**Purpose:**

The aim of this study is to systematically review the available literature on the performance of NLP in identifying the presence of an adenoma or a sessile/serrated adenoma in colonoscopy reports.

**Method:**

We performed a systematic review and meta-analysis according to PRISMA recommendations. A comprehensive literature query was conducted on MEDLINE, EMBASE, CINAHL, and CDSR, through July 2022. Studies were included if they evaluated the performance of NLP in extracting data from colonoscopy reports. Our primary outcome was the performance of NLP models in correctly identifying an adenoma reported in a colonoscopy report. Two authors independently screened studies and abstracted data using an *a priori* designed data collection form. We pooled the sensitivity and specificity of our primary outcome using a univariate analysis first, followed by a bivariate analysis. Using the open-source package ‘mada’ which is written in R, we generated a summary estimate and a summary receiver operating characteristic curve.

**Result(s):**

From the 1030 unique studies obtained from our literature search, 13 studies met the inclusion criteria. Eligible studies were used for our meta-analysis. In the univariate analysis, the pooled sensitivity and specificity for detecting an adenoma by the NLP systems was 0.978 (95% CI 0.938-0.992) and 0.997 (95% CI 0.984-0.999), respectively. Similarly, in univariate analysis, the pooled sensitivity and specificity for detecting a sessile/serrated adenoma by the NLP systems was 0.984 (95% CI 0.929-0.996) and 1.0 (95% CI 0.998-1.000), respectively. In the bivariate analysis, the summary estimates for the sensitivity and specificity of the NLP system in detecting an adenoma were 0.973 (95% CI 0.929-0.990) and 0.992 (95%CI 0.978-0.997) respectively. For detecting a sessile/serrated adenoma, the summary estimates for sensitivity and specificity were 0.964 (95% CI 0.895-0.988) and 0.998 (95% CI 0.995-0.999) respectively.

**Conclusion(s):**

NLP models have excellent performance in extracting quality metric data from colonoscopy reports. Based on the available literature, we suggest integration of NLP in quality improvement efforts in colonoscopy.

**Please acknowledge all funding agencies by checking the applicable boxes below:**

None

**Disclosure of Interest:**

None Declared

